# Cellulo-Cutaneous Erysipelas

**Published:** 1875-12

**Authors:** J. M. Williamson

**Affiliations:** Evansville, Indiana


### CELLULO-CUTANEOUS ERYSIPELAS.

BY J. M. WILLIAMSON, EVANSVILLE, INDIANA.

I herewith send you report of a case of cellulo-cutaneous erysipelas which recently came under my notice, in which I used as a trial application the ung. oxid. zinc benz. with marked success.

Mrs. D., of this city, a lady of about 37 years, mother of seven children, of full habit, was attacked with cellulo-cutaneous erysipelas on the 25th of September, and sent for me.

The disease had made its appearance first on the hands, showing itself a few hours afterwards on the face. At the time of my visit the hands were very much swollen, and the face so much so as to completely close both eyes. The pungent, burning pain in the affected parts caused the patient great distress, and there was considerable constitutional disturbance.

She informed me that this was the third attack she had experienced within the last two years, but that this, she thought, had come on more violently than either of the preceding ones. The attack last before the present one, from which she suffered some ten months ago, had confined her to her room for several weeks, and she was now evidently much discouraged at the thought of being disabled for so long a time again.

The physician who had previously attended her, she informed me, had applied to the affected parts lead lotion, alternated with tr. ferri muri. and tr. iodine, and she objected now to being, even temporarily, disfigured by applications which seemed to her to offer so little relief, if it was possible to employ any other mode of treatment. I directed the following prescription, and left, to return next morning:

℞—Liquor mag. citratis (to be taken at once), . . ℥xii.

Tr. ferri muriatis, . . . . . . . . . . . ℥ii.

Glycerine, . . . . . . . . . . . . . . . ℥i.

M. Sig. Teaspoonful every two hours during the day; hands and face to be enveloped in soft cotton batting.

Visited patient again the next morning, Sept. 26th, and was notified that her bowels had been fairly moved the day before from the magnesia, but that she had passed a restless day and night. She was still complaining of the "smarting" in her face and hands, which, as she expressed it, was "intolerable." On removal of the cotton batting, I found that the eye lids were discharging a sero-purulent fluid, and that large blebs, containing the same kind of fluid had formed on the palm surface of the hands and fingers. These I opened with a fine needle. I then had the entire face and the hands thickly coated over with ung. oxid. zinc benz., and the cotton reapplied, and ordered a continuance of the tr. ferri muriatis as prescribed the day before. Bromide potassium, with chloral hydrate, each grs. x, were directed to be given at bed-time should the patient continue restless. On my return on the morning of the 27th, patient expressed herself as feeling much better; had slept well through the night without the use of the sedative left for her; said the ointment had relieved the burning sensation shortly after being applied, and that she had suffered but little from this since. I ordered the ointment to be applied again as before, and continued the tr. ferri internally. Next morning, 28th, patient was in about the same condition as when I saw her last; but bowels not having moved since the 25th, a siedlitz was directed, which had the desired effect. Quinia in three grain doses was now added to the iron prescription, which was continued, and the ointment freshly applied. On the 29th patient was very much better; could now see a little out of both eyes, so much was the œdema reduced.

Treatment continued; 30th, still improving; treatment as before; diet of soft boiled eggs, milk, rich soups, etc., prescribed. October 1st, patient able to sit up in her room; constitutional disturbances entirely disappeared; swelling much reduced; burning sensation, which had not been troublesome since first application of ointment, now not felt at all; kept up the iron and quinia internally, and ointment with cotton covering locally, until October 5th, when treatment was suspended, the patient being now perfectly well, and delighted at her rapid recovery, and, as she said, quite so at not being left with a stained face as on previous occasions.

I do not remember ever to have seen the ung. oxidi zinc benz. recommended in erysipelas, but in this instance it certainly proved a most successful topical agent.

